# Response to Dengue virus infections altered by cytokine-like substances from mosquito cell cultures

**DOI:** 10.1186/1471-2180-10-290

**Published:** 2010-11-16

**Authors:** Nipaporn Kanthong, Chaowanee Laosutthipong, Timothy W Flegel

**Affiliations:** 1 Dept. Biotechnology, Faculty of Science and Technology, Rajamangala University of Technology Tawan-ok, Sriracha, Chonburi 20110, Thailand; 2 Dept. Biotechnology, Faculty of Science, Mahidol University, Rama 6 Road, Bangkok 10400, Thailand; 3 Centex Shrimp, Faculty of Science, Mahidol University, Rama 6 Road, Bangkok 10400, Thailand; 4 National Center for Genetic Engineering and Biotechnology (BIOTEC), National Science and Technology Development Agency, Klong 1, Klong Luang, Pratum Thani 12120, Thailand

## Abstract

**Background:**

With both shrimp and commercial insects such as honey bees, it is known that stable, persistent viral infections characterized by absence of disease can sometimes shift to overt disease states as a result of various stress triggers and that this can result in serious economic losses. The main research interest of our group is to understand the dynamics of stable viral infections in shrimp and how they can be destabilized by stress. Since there are no continuous cell lines for crustaceans, we have used a C6/36 mosquito cell line infected with Dengue virus to test hypotheses regarding these interactions. As a result, we accidentally discovered two new cytokine-like substances in 5 kDa extracts from supernatant solutions of acutely and persistently infected mosquito cells.

**Results:**

Naïve C6/36 cells were exposed for 48 h to 5 kDa membrane filtrates prepared from the supernatant medium of stable C6/36 mosquito cell cultures persistently-infected with Dengue virus. Subsequent challenge of naïve cells with a virulent stock of Dengue virus 2 (DEN-2) and analysis by confocal immunofluorescence microscopy using anti-DEN-2 antibody revealed a dramatic reduction in the percentage of DEN-2 infected cells when compared to control cells. Similar filtrates prepared from C6/36 cells with acute DEN-2 infections were used to treat stable C6/36 mosquito cell cultures persistently-infected with Dengue virus. Confocal immunofluorescence microscopy revealed destabilization in the form of an apoptosis-like response. Proteinase K treatment removed the cell-altering activities indicating that they were caused by small polypeptides similar to those previously reported from insects.

**Conclusions:**

This is the first report of cytokine-like substances that can alter the responses of mosquito cells to Dengue virus. This simple model system allows detailed molecular studies on insect cytokine production and on cytokine activity in a standard insect cell line.

## Background

It is well known that stable, persistent viral infections can be maintained in insect cell cultures and that such cultures often show no adverse signs of infection [1-6]. This phenomenon has been most studied in arboviruses such as Dengue virus that are carried by insect host vectors as innocuous infections, but cause disease in target vertebrate hosts. In fact, persistent, innocuous, viral infections appear to be common in insects and crustaceans as single infections or dual to multiple co-infections [7,8]. With both shrimp and commercial insects such as honey bees, it is known that these stable, persistent infection states characterized by absence of disease can sometimes shift to overt disease states as a result of various stress triggers [9-13] and that this can result in serious economic losses [7,14,15]. Thus, the main research interest of our group focuses on understanding the dynamics of single to multiple, persistent viral infections in shrimp and how environmental conditions or other stress can sometimes destabilize them. Since no continuous cell lines have ever been successfully developed for crustaceans, we have had to turn to continuous insect cell lines and insects to try to understand the dynamics of these interactions [6,16].

During the course of establishing C6/36 mosquito cell cultures persistently infected with Dengue virus, we accidentally discovered that cell-free supernatant solutions from these cultures could reduce and delay the onset of cytopathology in naïve C6/36 cells newly challenged with Dengue virus. Conversely, cell-free supernatant solutions from acutely infected cultures were capable of destabilizing persistently-infected cultures in a manner similar to the destabilization that occurs in shrimp and insect populations. Here we describe the relevant experiments and show that the active factors in the cell-free supernatant solutions are probably small polypeptides with cytokine-like activity.

## Results and discussion

### Persistent Dengue virus infections

After primary challenge of naïve C6/36 cell cultures with DEN-2 followed by split-passage every 2 days, stable cultures persistently infected with DEN-2 were obtained with 100% DEN-2 positive cells, as previously described [6]. The growth rate of cultures persistently infected with DEN-2 did not differ significantly (p > 0.05) from that of uninfected cell cultures. The gross signs of DEN-2 infection declined with increasing passage number. From passage 15 onwards the cultures did not differ morphologically from naïve C6/36 cell cultures. However DEN-2 released into the culture medium could initiate acute DEN-2 infections in naïve cells, as previously reported [6]. Neither these preparations nor DEN-2 stock inoculum caused any changes when used to challenge cultures persistently infected with DEN-2.

### Filtrate from persistently infected cells protects naïve cells against DEN-2

**Figure 1:**
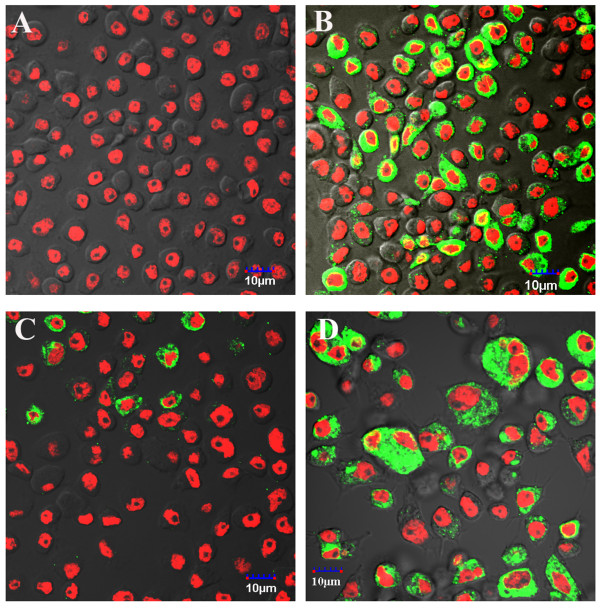
**C6/36 cells protected against DEN-2 by 5 kDa membrane filtrate from cell cultures persistently infected with DEN-2**. A = Unchallenged, naïve cells showing no immunofluorescence for DEN-2; B = Untreated, naïve cells challenged with DEN-2 and showing a high prevalence DEN-2 immunopositive cells (green) at 24 h post challenge; C = Naïve cells pre-exposed to the 5 kDa filtrate from C6/36 cells persistently infected with DEN-2 prior to DEN-2 challenge and showing few DEN-2 immunopositive cells at 24 h post challenge; D = Naïve cells treated with the wash from the upper side of the 5 kDa filter but **not **challenged with DEN-2 stock and showing a high prevalence of DEN-2 immunopositive cells at 24 h post challenge with viral particles in the supernatant solution from the persistently-infected culture. Green = anti-DEN and Red = pseudocolor for T0-PRO-3 iodide staining of DNA (nuclei).

Immunofluorescence assay using an antibody to DEN-2 envelope protein revealed that 48-h pretreatment of naïve C6/36 cells with the 5 kDa filtrate from cell cultures persistently infected with DEN-2 led to a significant reduction (p = 0.009) in the percentage of DEN-2 immunopositive cells (6 ± 5%) when compared to untreated cells after DEN-2 challenge (46 ± 2%) (Figure 1). These results were confirmed by using Vero cells to measure the DEN-2 titers in supernatant solutions from the treated insect cells. The titers were 2 × 10^6 ^+/- 0 at 24 h and 8 × 10^6 ^+/- 0 at 48 h for naive cells but 6 × 10^4 ^+/- 2 × 10^4 ^at 24 h and 3.2 × 10^3 ^+/- 2.4 × 10^3 ^at 48 h for filtrate-exposed cells (significant differences for both times at p = 0.001). To achieve the maximum reduction in numbers of immunopositive cells and the least cytopathology, it was necessary to pre-incubate the cells for 48 h prior to DEN-2 challenge. Exposure to the active preparation for periods less than 48 h was proportionally less effective in inducing resistance (not shown). The pre-incubation requirement suggested that reduction in severity of DEN-2 infection was induced in the challenged cells by an active factor(s) in the filtrate.

**Figure 2:**
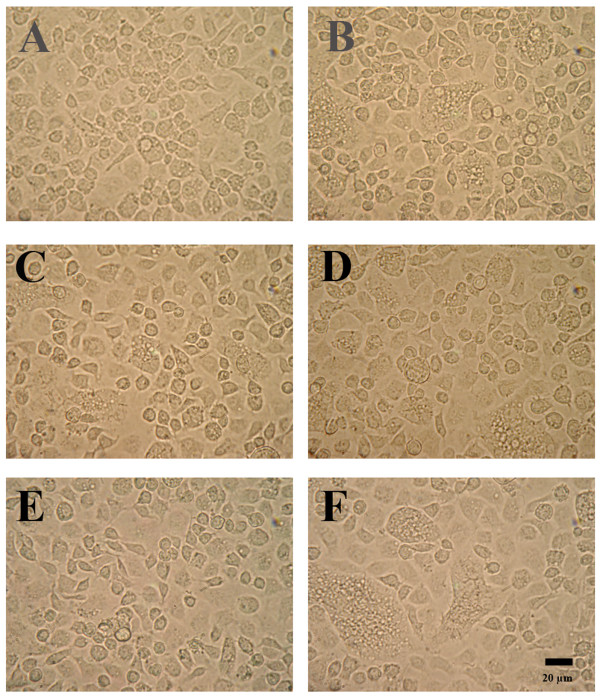
**Phase contrast photomicrographs of C6/36 cells at 2 days post-challenge with DEN-2**. (A) Unchallenged naïve control cells. (B) Untreated C6/36 cells challenged with DEN-2 stock and showing cytopathic, fused giant cells. (C) C6/36 cells pre-treated with the 5 kDa filtrate before challenge with the DEN-2 stock and showing fewer cytopathic, fused giant cells than the untreated cells in B. (D) C6/36 cells exposed to the whole supernatant solution from cultures persistently infected with DEN-2 and showing similar cytopathology to that in B. (E) C6/36 cells exposed to the 5 kDA filtrate (control not challenged with DEN-2) and showing no cytopathology (i.e., similar to A with no DEN-2 infection). (F) C6/36 cells exposed to the wash from the upper side of the 5 kDa membrane and showing the greatest number of cytopathic giant cells (i.e., more than that in B and similar to Figure 1D).

To confirm that the DEN-2 positive cells arose from challenge with the DEN-2 stock and not from virions in the 5 kDa filtrate, naïve C6/36 cells were exposed to the 5 kDa filtrate, to wash from the upper side of the 5 kDa membrane and to unfiltered supernatant solution from the culture from which the filtrate was derived (i.e., 19^th ^passage of a culture persistently infected with DEN-2) (Figure 2). After 2 days of incubation, phase contrast microscopy revealed that the wash from the upper side of the 5 kDa membrane resulted in the most severe cytopathology (i.e., many fused giant cells) in the naïve C6/36 cells (Figure 1D and Figure 2F), while exposure to the whole, unfiltered culture filtrate (Figure 2D) gave cytopathology similar to that produced by the DEN-2 stock (i.e., fewer fused giant cells)(Figure 2B). Pre-exposure of naïve C6/36 cells to the 5 kDa filtrate reduced the severity of Dengue infection (i.e., no fused giant cells) (Figure 2C) and exposure to the 5 kDa filtrate in the absence of DEN-2 challenge resulted in no cytopathology (Figure 2E), i.e., morphology similar to that of unchallenged, naïve cells (Figure 2A).

In summary, results from these tests indicated that 48 h pre-exposure of C6/36 cells to a low molecular weight substance(s) in a 5 kDa filtrate from persistently-infected cells was able to induce a protective response against DEN-2 virus infection in naïve cells. Molecules that have such activity can be called cytokines and we would like to coin the term "viprolaxikine" (derived from "a cytokine for viral prophylaxis") for the agent(s) discovered in this work.

To date, few cytokines have been described from insects or insect cells. Examples include a growth-blocking peptide present in hemolymph of larvae of the insect armyworm *Pseudaletia separata *parasitized by the wasp *Apanteles kariyai*. The growth-blocking peptide has repressive activity against juvenile hormone esterase [17]. Another growth-blocking peptide (GBP) from Lepidopteran insects regulates larval growth, cell proliferation, and immune cell (plasmatocyte) stimulation [18]. These cytokines belong to what is called the ENF multifunctional peptide family that is characterized by the unique ENF amino acid consensus sequence at their N termini [19]. One of these ENF peptides has been reported to be induced by viral infection in silkworms [20] and another from moth larvae has been reported to stimulate aggregation and directed movement of phagocytic hemocytes [21]. By contrast, the non-ENF cytokine, astakine was actually required for infectivity of white spot syndrome virus in haematopoietic cells of the freshwater crayfish, *Pacifastacus leniusculus *[22].

Another group of insect cytokine-like peptides that have antiviral activity are called alloferons [23]. These peptides are composed of 12-13 amino acids and they can stimulate natural cytotoxicity of human peripheral blood lymphocytes, induce interferon synthesis in mouse and human models, and enhance antiviral and antitumor activity in mice. Although the effect of these substances on insect cells has not been reported, it is possible that viprolaxikine may be an alloferon-like substance. If so, it would be the first alloferon-like substance reported to be produced in an insect cell culture rather than in whole insects. If so, this insect system might constitute a simple model for studying alloferon induction and alloferon control mechanisms in insect cells.

Another antiviral protein (AVP) has been described from C6/36 cells persistently infected with Sindbis virus [24]. It was purified to homogeneity and found to be a very hydrophobic peptide of 3200 kDa [25]. When only one clone (U4.4) of naïve C6/36 cells is exposed to AVP for 48 h, the cells not only became refractory to infection by Sindbis virus but also continuously produced AVP and remained refractory to Sindbis virus upon subsequent passage, i.e., they became permanently altered by a single exposure to AVP. AVP had no protective activity against Sinbis virus in BHK-21 mammalian cells [26] and the actual amino acid sequence has not been reported. The requirement for 48 h pre-exposure to obtain protection against Sindbis virus is similar to the requirement of pre-incubation with viprolaxikine for DEN-2 protection in C6/36 cells. An antiviral substance similar to AVP was reported from mosquito cells infected with Semliki Forest virus (SFV) (family *Alphaviridae*) but not from mosquito cells infected with encephalitis virus (family *Flaviviridae*) or Bunyamwera virus (family *Bunyaviridae*), leading to the suggestion that AVP-like substances were unique to viruses in the family *Alphaviridae *[27]. Thus, viprolaxikine has some similarities to AVP in terms of small size and pre-exposure requirement for activity, but it also differs in arising from cells infected with a virus from the family *Flaviviridae*. Since the structure of AVP and viprolaxikine are still unknown their relationship to each other and to ENF peptides and alloferons is currently unknown.

### Filtrate from acutely infected cells destabilizes persistently infected cells

**Figure 3:**
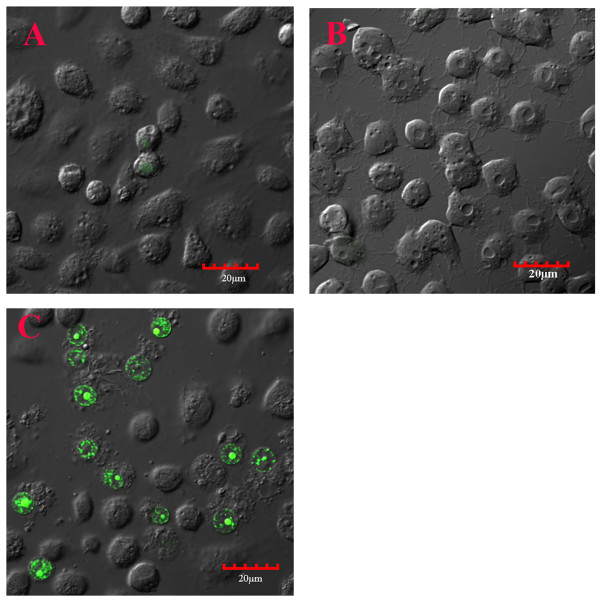
**Apoptosis induction by 5 kDa membrane filtrate in cultures persistently infected with DEN-2**. A = Untreated naïve C6/36 control cells; B = C6/36 cells from a culture persistently infected with DEN-2; C = As in B except treated with the 5 kDa filtrate from the supernatant of C6/36 cells acutely infected with DEN-2 and showing nuclei with positive immunoflurescence (green) for the apoptosis marker YO-PRO-1 iodide.

When C6/36 cells persistently-infected with DEN-2 (19^th ^passage) were exposed to cell-free filtrate from acutely infected cells (i.e., naïve cells 2 days post challenge with DEN-2 stock) a confocal immunofluorescence assay for apoptosis-like activity revealed positive signals (32 ± 12% of cells) but none in untreated cells at 24 h post exposure (Figure 3C). The YO-PRO-1 positively-stained cells increased with time and at 3-5 days post-exposure some CPE was seen, but this was less than that observed when naïve cells were challenged with DEN-2 stock. In addition, split-passage of the filtrate-exposed cultures led to more rapid return to normal cell morphology than occurred with DEN-2-challenged, naïve cells.

No apoptosis activity was detected in control cell cultures persistently infected with DEN-2 (19^th ^passage) but not exposed to filtrate (Figure 3B). Nor were there any apoptosis-positive cells in persistently-infected cells exposed to 5 kDa membrane filtrates from naïve cells (image the same as that in 3B). The complete absence of apoptosis in these persistently infected cells contrasted with a very small number of weakly immunopositive cells in untreated naïve C6/36 cell cultures (Figure 3A), indicating a low level of apoptosis. This is not uncommon, since apoptosis is a normal process for maintenance of homeostasis and elimination of occasional aberrant cells [28]. For example, low levels of apoptosis have been previously reported for normal, uninfected C6/36 control cells in experiments with Sindbis virus [29]. Absence of any apoptosis in the persistently-infected cell cultures may indicate that it is being positively suppressed.

The induction of apoptosis by addition of a filtrate containing a low molecular weight agent(s) to grossly normal, stable cultures of mosquito cells persistently infected with DEN-2 constitutes a process of destabilization of the persistent infection and at least partial reversion to a diseased state with reoccurrence of pathology seen when naïve C6/36 cells are first exposed to DEN-2 prior to serial passage. This resembles the situation that occurs when innocuous, persistent, viral infection states in shrimp and insects are shifted to disease states by stress triggers. It has been reported that massive apoptosis called kakoapoptosis [8,30] occurs in moribund shrimp infected with white spot syndrome virus (WSSV) [31,32] and yellow head virus [33]. Our results raise the possibility that such apoptosis may be mediated by a low molecular weight cytokine-like agent(s) that could be triggered by various types of stress in cells persistently infected with viruses and could be referred to as apinductokine (i.e., apoptosis inducing cytokine). For example, mammalian tumor necrosis factor (TNF) is the prototypic member of a family of cytokines that interact with a large number of receptors and may induce apoptosis [34]. Insects have been reported to have homologues of TNF (e.g., Eiger) [35-38] and to TNF receptors (e.g. Wengen) [39,40]. There are recent indications that they may be related to stress-induced apoptosis in insects via the JNK pathway [41,42]. Given that the cytokine-like substance described herein is very much smaller than even the soluble form of Eiger, it is probably a distinct identity that may function via a receptor distinct from Wengen.

In any case, this cytokine-like model for destabilization of C6/36 cells persistently infected with DEN-2 provides the first opportunity for detailed analysis of the underlying molecular mechanisms both for production of this cytokine and for its induction of apoptosis using such tools as gene expression analysis by suppression subtractive hybridization.

### Viprolaxikine activity removed by proteinase-K treatment

**Figure 4:**
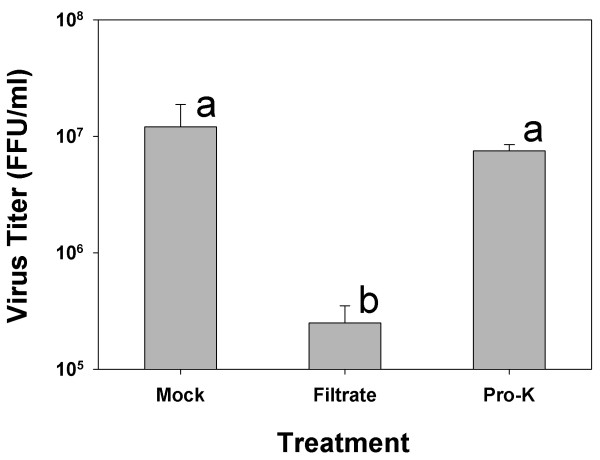
**Removal of protection against DEN-2 by filtrate treatment with proteinase K**. Bar Mock = Mock-treated, naïve cells exposed to DEN-2 stock; Bar Filtrate = Cells pre-exposed for 48 h to the 5 kDa filtrate from cultures persistently infected with DEN-2 (passage 16) and showing more than 2 logs decrease in DEN-2 titer; Bar Pro-K = Cells pre-exposed for 48 h with proteinase-K-treated 5 kDA filtrate and showing a major recovery in DEN-2 titer. Bars indicate mean titers ± SD for 3 replicates and those labeled with different letters are significantly different (p < 0.05) while those with the same letter are not (p > 0.05).

Trials on proteinase treatment of filtrates were carried our using Vero cells to measure the DEN-2 titers in the supernatant solutions of naïve C6/36 cells pre-exposed to filtrates prior to challenge with the DEN-2 stock inoculum. Results (Figure 4) showed that mock-treated naïve C6/36 cells (positive control) yielded high titers (mean 1.2 × 10^7 ^± 6.7 × 10^6 ^FFU/ml) while cells pre-exposed to filtrate yielded significantly (p = 0.039) lower titers (mean 2.5 × 10^5 ^± 1.0 × 10^5^), and cells pre-exposed to proteinase-K-treated filtrate yielded titers (mean 7.5 × 10^6 ^± 1.0 × 10^6^) not significantly different (p = 0.2) from the positive control. Results were similar whether proteinase-K activity was removed after filtrate treatment by heating plus 5 kDa filtration or by 5 kDa filtration only. Since, proteinase-K treatment almost completely removed protection and restored the titer of the DEN-2 stock solution, it was concluded that viprolaxikine was most likely a small polypeptide.

### Apinductokine activitiy removed by Proteinase-K treatment

**Figure 5:**
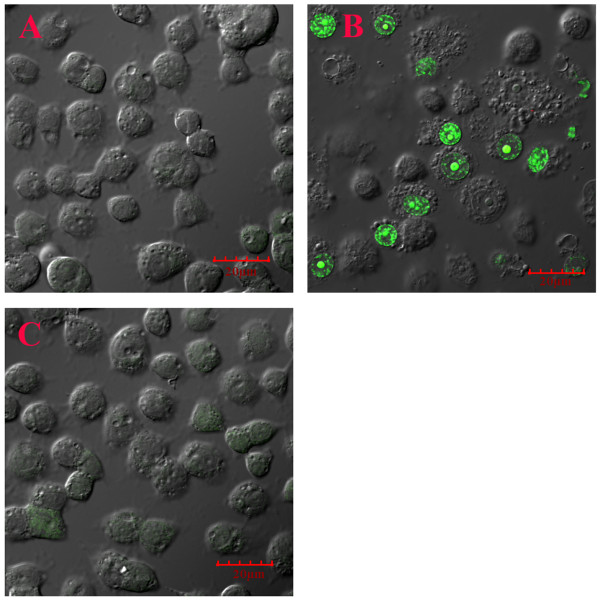
**Photomicrographs showing removal of apoptosis induction activity by proteinase K treatment**. A = Untreated, cells persistently infected with DEN-2 (cf Fig. 3A); B = Positive immunofluorescence for apoptosis marker (green) in cells persistently infected with DEN-2 and exposed to untreated 5 kDa filtrate from C6/36 cells acutely-infected with DEN-2; C = As in B, but with proteinase-K treatment and showing little positive fluorescence (green) for the apoptosis marker.

Proteinase-K treatment of 5 kDa membrane filtrates from C6/36 cultures acutely infected with DEN-2 removed their ability to induce apoptosis in C6/36 cells persistently infected with DEN-2 (Figure 5). As with viprolaxikine, apinductokine inactivation occurred whether proteinase-K activity was removed from the treated filtrate by heating plus 5 kDa filtration or by 5 kDa filtration only. These tests indicated that apinductokine was also a small polypeptide.

## Conclusion

In conclusion, this communication has revealed that extracts from C6/36 cell cultures infected with Dengue virus contain previously unknown cytokine-like substances that can alter the host insect cell response to Dengue virus. It is the first report of an antiviral substance induced in insect cells by infection with a virus in the family *Flaviviridae*. The fact that the cell sources and activities of the substances differed and that their activities were removed by treatment with proteinase-K suggested that at least two different, low molecular-weight polypeptides were responsible, one for protection of naïve cells against DEN-2 infection and the other for induction of apoptosis in C6/36 cells persistently infected with DEN-2. Further work is needed to characterize these cytokine-like substances (including molecular structure) to allow comparison with other low molecular weight polypeptides, to study their mechanism of action and to test their range of activities with several viruses and cell types.

## Methods

### Insect cell lines and viral inoculum

*Aedes albopictus *C6/36 cells (a single cell-type clone obtained from the American Type Culture Collection under catalogue number CRL-1660) were grown in Leibovitz's (L-15) medium containing 10% heat-inactivated fetal bovine serum (FBS), 10% tryptose phosphate broth (TPB) and 1.2% antibiotic (Penicillin G and Streptomycin).

Dengue serotype 2 virus (DEN-2)(NGC strain) used in this work was obtained from the US Armed Forces Research Institute of Medical Sciences (AFRIMS), Bangkok through the courtesy of Ananda Nisalak and was stored in 20% fetal bovine serum at -80°C at the Division of Medical Molecular Biology, Office of Research and Development, Faculty of Medicine, Siriraj Hospital, Mahidol, University, Bangkok. After thawing at room temperature, the stock was used as inoculum for monolayers of naïve C6/36 cells in Leibovitz's (L-15) medium containing 1% heat-inactivated fetal bovine serum (FBS), 10% tryptose phosphate broth (TPB) and 1.2% antibiotic (Penicillin G and Streptomycin). At days 5-7 after challenge, the supernatant solution was removed and used as inoculum for subsequent trials.

### Naïve C6/36 cells challenged with Dengue virus

Culture plates (6-well, Costar, Corning) were seeded with C6/36 cells at a density 10^6 ^cells/well and incubated for 24 h at 28°C to produce confluent monolayers. The cell monolayers were then challenged with DEN-2 at a multiplicity of infection (MOI) of 0.1. After incubation for 2 h with gentle shaking at room temperature, the medium was removed and fresh medium containing 2% FBS was added for further incubation at 28°C.

### Persistent infection of C6/36 cells with Dengue virus

Persistent infections of DEN-2 in C6/36 cells were achieved as previously described [6]. Briefly, after 2 days incubation post DEN-2 challenge (acute infections in C6/36 cells), the supernatant solution was removed and cells were suspended by knocking in L-15 containing 10% FBS at 1:3 dilution and transferred to a new culture well at 1/2 density for 2-days cultivation (to full confluence) before repeating the decantation, suspension, dilution and transfer process sequentially at 2 day intervals to establish persistently infected cultures. Three replicates were done in 6 well plates at 2 day intervals. Mock-infected cells were run in parallel to the viral infected cells to serve as negative controls.

### Preparation of cell and virus free culture filtrates

Culture supernatant solutions (4 ml) from cultures acutely infected or persistently infected with DEN-2 were clarified by centrifugation at 2000 × g for 5 min. The supernatant was transferred to an Amicon Ultra filter unit (Millipore) containing a cellulose, low-binding membrane with a molecular weight cut-off of 5 kDa. The ultrafiltration device was centrifuged at 4000 × g for 25 min to produce a filtrate that consisted of substances that could pass the 5 kDa molecular weight cut-off. These filtrates were collected and stored at -20°C.

### Immunofluorescent staining for confocal microscopy

For DEN-2 detection, cells were fixed with 4% formaldehyde in PBS for 15 min, washed twice with PBS, permeabilized with 0.1% Triton X-100 for 5 min and blocked with PBS containing 10% FBS. Cells were incubated for 1 hour with 3H5 monoclonal antibody against DEN-2 virus envelope protein followed by incubation for 30 min. with 1:500 dilution of fluorophore-labeled secondary antibody conjugate (Alexa Flour 488 goat anti-mouse IgG, A-11001, from Molecular Probes) directed against the primary antibody. They were then washed with PBS before analysis. To-Pro-3 iodide (T-3605, Molecular Probes) was used for nucleic acid counterstaining. Immunofluorescent-stained cells were analyzed by fluorescence microscopy and confocal laser microscopy (FV1000, Olympus).

For detection of apoptosis activity, live cells were removed from cultures and washed twice with PBS. They were incubated for 15 min with YO-PRO-1 iodide (Y3603, Molecular Probes) as a marker for apoptosis. It has been used previously as a marker for apoptosis in mosquitoes [43,44]. Immunofluorescent-stained cells were analyzed by fluorescence microscopy and confocal laser microscopy (FV1000, Olympus) within 30 min.

To determine the percentage of immunopositive cells, separate confocal photomicrographs from each test group were counted to obtain a total cell count of not less than 300. The percentage of immuopositive cells in each photomicrograph was then determined and the mean plus or minus 1 standard deviation of the mean (SD) was calculated for the photomicrographs of each test group. The Student t test (SigmaStat 3.5, Systat Software Inc., Chicago) was used for pair-wise group comparisons and differences between groups were considered significant when p ≤ 0.05.

### DEN-2 titer measurement using Vero cells

The DEN-2 stock solution and C6/36 cell-culture supernatants were subjected to standard assays of Dengue virus titers by measurement of focal forming units (FFU) per ml in Vero cell monolayers [6].

### Proteinase-K treatment of 5 kDa filtrates

Filtrates of cell free supernatants from passage 16 (P16) of C6/36 cell cultures persistently-infected with DEN-2 were treated with Proteinase-K enzyme (Invitrogen) for 30 min at 37°C. Controls consisted of filtrates from P16 of naïve C6/36 cells treated in the same manner. In initial tests, the enzyme was inactivated by heating at 90°C for 5 min followed by elimination via membrane filtration with a 5 kDa cutoff, as described above. In subsequent tests, the enzyme was eliminated simply by membrane filtration (5 kDa cutoff). C6/36 cells or Vero cells were pre-exposed to enzyme-treated filtrates and untreated control filtrates for 48 h before challenge with DEN-2 stock virus. Parallel tests included untreated, naïve cells challenged or not with DEN-2 stock (as above), naïve cells challenged with whole, untreated supernatant from passage 16 (P16) of C6/36 cultures persistently infected with DEN-2, and naïve cells challenged with the wash from the upper side of the 5 kDa membrane filter.

## Authors' contributions

NK and CL participated in the study design and the cell culture work, did the immunohistochemistry work, drafted the original manuscript and assisted in manuscript completion. TWF participated in the design and coordination of the work and took major responsibility for writing the manuscript. All authors read and approved the final manuscript.
